# Provision of bystander CPR for out-of-hospital cardiac arrest in the Middle East: a retrospective gender-based analysis

**DOI:** 10.1186/s12245-023-00537-6

**Published:** 2023-09-26

**Authors:** Emad Awad, Guillaume Alinier, Hassan Farhat, Niki Rumbolt, Adnaan Azizurrahman, Buthaina Mortada, Rakan Shami

**Affiliations:** 1 College of Health Science, University of Doha for Science and Technology, Doha, Qatar; 2 BC RESURECT: Department of Emergency Medicine, University of British Columbia, Vancouver, BC Canada; 3 Department of Emergency Medicine, School of Medicine, University of Utah, Salt Lake, UT USA; 4 Hamad Medical Corporation Ambulance Service (HMCAS), Hamad Medical Corporation, Doha, Qatar; 5 School of Health and Social Work, University of Hertfordshire, Hatfield, UK; 6 Weill Cornell Medicine – Qatar, Doha, Qatar; 7 Faculty of Health and Life Sciences, Northumbria University, Newcastle, UK; 8 Faculty of Medicine “Ibn El Jazzar”, University of Sousse, Sousse, Tunisia; 9 Faculty of Sciences, University of Sfax, Sfax, Tunisia

**Keywords:** Cardiac arrest, Cardiopulmonary resuscitation, Gender differences, Middle East

## Abstract

**Background:**

Previous studies conducted in North America, Europe, and East Asia (Liu et al., EClinicalMedicine 44:101293, 2022; Matsui et al., JAMA Netw Open 2:e195111, 2019; Awad et al., J Am Coll Emerg Physicians Open 4:e12957, 2023; Yoon et al., Prehosp Emerg Care :1–7, 2022) reported gender disparities in the provision of bystander CPR for patients with out-of-hospital cardiac arrest (OHCA). However, it remains unknown whether similar disparities exist in the Middle Eastern and Gulf regions. The primary objective of this study is to evaluate gender differences in the provision of bystander CPR for patients with OHCA in Qatar.

**Methods:**

Retrospective analysis of data obtained from Hamad Medical Corporation OHCA registry in the State of Qatar (2016–2022). We included adults with non-traumatic and EMS-attended OHCA. We used multilevel logistic regression to examine the association between gender and provision of bystander CPR.

**Results:**

In total, 4283 patients were included. Of those, 3414 (79.7%) were males, 1639 (38.3%) arrested in public locations, and 1463 (34.2%) received bystander CPR. Unadjusted comparisons showed that females were significantly older than males (mean age: 62.2 vs. 52.7). Females had a lower proportion of OHCA occurring in public locations (15.1% vs. 44.2%) and a lower proportion of shockable rhythm (11.9 vs. 27.5%). Regarding the outcome variable (provision of bystander CPR), the unadjusted analysis showed that the proportion of females who received bystander CPR was lower than that of males (29.2% vs. 35.4%, *p* < 0.001). However, after adjustment, we found no significant difference in provision of bystander CPR by gender (adjusted OR female vs. male 0.99, 95% CI 0.84–1.20, *p* = 0.97). In the subgroup who arrested in public locations, the analysis revealed females had greater odds of receiving bystander CPR (adjusted OR female vs. male 1.47, 95% CI 1.10–1.82, *p* = 0.04).

**Conclusions:**

Overall, bystander CPR was less common in female gender; after adjustment for other covariates, including arrest location, we found no significant gender differences in provision of bystander CPR. We also observed that females were found to have a lower incidence of cardiac arrest in public locations. Nevertheless, if females were to experience cardiac arrest in a public location, they would be more likely to receive CPR. Further research is required to explain the observed differences in provision of bystander CPR.

## Introduction

Out-of-hospital cardiac arrest (OHCA) is a serious life-threating condition that poses a significant global health concern. The incidence rate of OHCA among adults worldwide is an average of 55 cases per 100,000 person-years [1]. OHCA victims need immediate interventions for optimal outcomes, including achieving return of spontaneous circulation (ROSC) and survival to hospital discharge [2, 3]. One of the most important interventions for OHCA is providing the victims with Cardio-Pulmonary resuscitation (CPR) [4–7]. Prompt initiation of CPR is crucial to maintain blood flow to the brain and other vital organs while awaiting the arrival of emergency medical services (EMS) personnel. A patient suffering from OHCA is approximately twice as likely to survive when immediate CPR is provided while waiting for EMS to arrive compared to patients not receiving CPR [6, 8].

Previous research has shown gender disparities in the OHCA interventions, including the provision of bystander CPR [9–13]. Previous studies have consistently reported that females receive bystander CPR less frequently than males [9–16]. Liu et al. recently analyzed data on 56,192 cases of OHCA in 13 Asian countries to examine if there is an association between gender and provision of bystander CPR. They found that female gender was associated with a lower odds of bystander CPR in public OHCA (OR 0.89, CI 0.70–0.99). Similar results have been reported in studies conducted in Europe [14, 17] and North America [7, 12, 18].

While previous studies conducted in North America [7, 11, 15, 18, 19], Europe [14, 17], and East Asia [10, 16, 20, 21] found gender disparities in the provision of bystander CPR, it remains unknown whether similar disparities exist in the Middle Eastern and Gulf regions. The primary objective of this study is to evaluate gender differences in the provision of bystander CPR for patients with OHCA in Qatar.

## Methods

### Design and settings

This was an observational retrospective analysis of data obtained from Hamad Medical Corporation (HMC) OHCA registry in the State of Qatar. Qatar is a Middle Eastern country located on the northeastern coast of the Arabian Peninsula with a population of 3,005,000 people [22]. The State of Qatar is characterized by cultural traditions and social norms that influence aspects of life. Qatar’s socio-cultural environment places a strong emphasis on family privacy and adherence to traditional gender roles. In the Middle East and Arab countries, including Qatar, women have traditionally held significant roles within the private, family-centric sphere, where the household serves as the focal point of social life. In terms of the proportion of women in the workforce, it tends to be relatively lower compared to Western societies [23, 24].

HMC is the main provider of healthcare in Qatar [25]. For pre-hospital health emergencies, Qatar’s diverse population is primarily served by the lone governmental pre-hospital EMS; Hamad Medical Corporation Ambulance Service (HMCAS). HMCAS personnel respond to 999 emergency calls across Qatar by offering comprehensive emergency and non-emergency services. Once a call for service (CFS) is made, emergency medical dispatchers (EMDs) at the National Command Center use specialized software to ensure timely dispatch of the nearest emergency response unit (ERU), which may consist of various combinations of medical professionals such (ambulance paramedics and critical care paramedic). Upon arrival, paramedics administer emergency care and facilitate patient transfer to the most appropriate healthcare facility [26].

The HMC OHCA registry was designed as a population-based registry of data collected on all EMS-attended OHCA cases occurring in all regions in Qatar. The data were collected by the HMCAS and eight public hospitals affiliated with HMC [25, 27]. Ethical approval for this study was obtained from HMC - Medical Research Center (MRC) Ethics Board.

### Study population

We created an analytic dataset from HMC OHCA Registry. We included all adult males and females 18 years and older who experienced non-traumatic OHCA and were treated by EMS. We excluded patients < 18 years, patients with traumatic arrest, those pronounced dead on EMS arrival, and patients missing data on gender or other variable required for the analysis.

### Variable of interest

The independent variable was gender (male vs. female), and the main outcome of interest was provision of bystander CPR. Based on the Utstein definitions, bystander CPR was defined as “CPR performed by layperson before the EMS arrival” [28].

### Statistical analysis

We calculated summary statistics for each baseline characteristic. Continuous variable (age) was summarized using mean and standard deviation as it was approximately normally distributed, and categorical variables were summarized using frequencies and percentages. To examine the association between gender and baseline characteristics, we used chi-square test for categorical variables and Student’s *t*-test for the continuous variable. Specifically, we used chi-square tests to compare provision of bystander CPR and other baseline characteristics between males and females. We assessed both chi-square and Student’s *t*-tests at a 5% level of significance.

To examine the adjusted relationship between gender and the provision of bystander CPR, we fit multivariable logistic regression model with gender as the independent variable and provision of bystander CPR as the dependent variable. We started the regression analysis by including only gender in the model, and then, we used forward variable selection technique to add other covariates. We adjusted for the covariates known to be associated with OHCA outcomes, including age (per year), location of arrest (public vs private location), witness status (witnessed vs. unwitnessed), initial cardiac rhythm (shockable vs non-shockable), and period of arrest (COVID-19 pandemic vs. non-pandemic period). For the last variable, we categorized OHCA cases that occurred between March 11, 2020, and September 15, 2022, as pandemic cases, while the cases that occurred before or after this time frame were classified as non-pandemic. We incorporated interaction terms (age X gender) to test significance. The interaction was not included in the model if it was not statistically significant. We conducted the multivariable analysis again for subgroup of the cohort who arrested in a public location. We assessed the absence of multicollinearity among the variables by computing the Variance Inflation Factor (VIF) [29]. All analyses were performed using IBM SPSS (version 29) [Computer software]. Armonk, NY. (2021).

## Results

### Baseline characteristics and unadjusted analysis

**Fig. 1:**
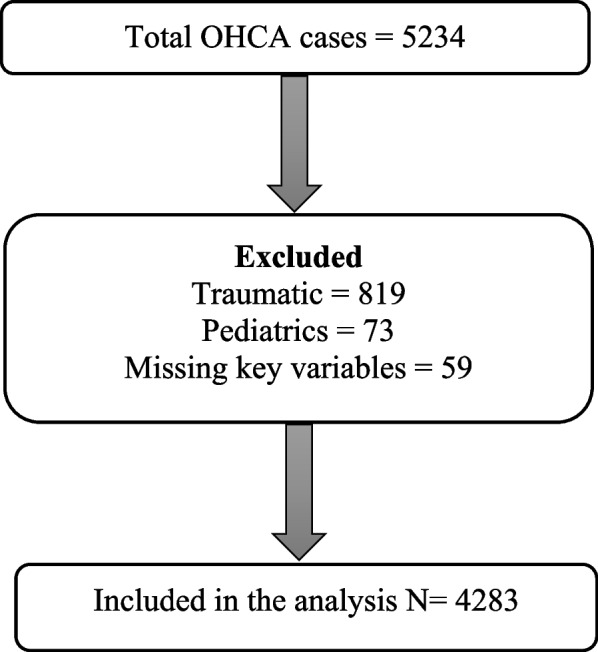
Study flow chart

We reviewed 5234 OHCA cases. Of these, we excluded 819 patients who experienced traumatic arrest and 73 patients who were under 18 years of age. We additionally excluded 59 cases missing data on gender or outcome variables. Therefore, the analytic dataset included 4283 adult, non-traumatic, EMS attended OHCA cases (Fig. 1). The mean age of the study cohort was 56.0 (17.4 SD). Of the total, 3414 (79.7%) were males, 1639 (38.3%) arrested in public locations, and 1463 (34.2%) received bystander CPR. Table 1 shows the descriptive statistics for the study variables overall and stratified by gender.

**Table 1:** Baseline characteristics stratified by gender

Variable	Total *N* = 4283	Female 869 (20.3%)	Male 3414 (79.7%)	*P* value
**Age**	56.0 ± 17.4	62.2 ± 19.1	52.7 ± 16.4	< 0.001
**Arrest location**				
Private	2644 (61.7%)	738 (84.9%)	1906 (55.8%)	< 0.001
Public	1639 (38.3%)	131 (15.1%)	1508 (44.2%)	
**Witnessed status**				
EMS^a^-witnessed	674 (15.7%)	169 (19.4%)	505 (14.8%)	< 0.001
Bystander-witnessed	3609 (84.3%)	700 (80.6%)	2909 (85.2%)	
**Bystander CPR**^**b**^				
Not provided	2820 (65.8%)	615 (70.8%)	2205 (64.6%)	< 0.001
Provided	1463 (34.2%)	254 (29.2%)	1209 (35.4%)	
**Initial rhythm**				
Non-shockable	3241 (75.7%)	766 (88.1%)	2475 (72.5%)	< 0.001
Shockable	1042 (24.3%)	103 (11.9%)	939 (27.5%)	
**Arrest period**				
Non-pandemic	2404 (56.1%)	501 (57.7%)	1903 (55.7%)	0.320
Pandemic	1879 (43.9%)	368 (42.3%)	1511 (44.3%)	
**ROSC**^**c**^				
Not achieved	3027 (70.7%)	610 (70.2%)	2417 (70.8%)	0.380
Achieved	1256 (29.3%)	259 (29.8%)	997 (29.2%)	

^a^*EMS* Emergency medical service

^b^*CPR* Cardio-pulmonary resuscitation

^c^*ROSC* Return of spontaneous circulation

Unadjusted comparisons between males and females showed that females were significantly older than males (mean age: 62.2 vs. 52.7). Females had a lower proportion of OHCA occurring in public locations (15.1% vs. 44.2%) and a lower proportion of shockable rhythm as a first cardiac arrest rhythm (11.9 vs. 27.5%). The unadjusted analysis also revealed no statistically significant gender differences in achieving ROSC (29.2% vs. 29.8%, *p* = 0.32). Regarding the outcome variable (provision of bystander CPR), the unadjusted analysis showed that the proportion of females who received bystander CPR was lower than that of males (29.2% vs. 35.4%, *p* < 0.001) (Table 1).

### Adjusted analysis result

The crude OR of provision of bystander CPR (female vs. male) was 0.75, 95% CI 0.64–0.88. While the unadjusted analysis revealed females were less likely to receive bystander CPR than males, the adjusted analysis showed no significant difference in provision of bystander CPR by gender (adjusted OR female vs. male 0.99, 95% CI 0.84–1.20, *p* = 0.97) (Table 2). The final model included gender, age, arrest location, witness status, period of arrest, and initial rhythm (Table 2). Our analysis revealed that all the VIF values were below 2.0, which indicates the absence of significant multicollinearity among the independent variables [29].

**Table 2:** Logistic regression: association between gender and bystander CPR (*N* = 4283)

Variable	OR^a^	(95% CI^b^)	*P-*values
Female gender (crude)	0.75	0.64–0.88	< 0.001
Female gender (adjusted)	0.99	0.84–1.20	0.974
Age	1.01	0.99–1.01	0.163
Public location	1.88	1.64–2.17	< 0.001
Witnessed arrest	4.58	3.62–5.80	< 0.001
Initial rhythm	1.80	1.52–2.11	< 0.001
Non-pandemic period	1.24	1.08–1.40	< 0.001

^a^*OR* Odds ratio

^b^*CI* Confidence intervals

We performed multivariable logistic regression analysis again for the subgroup of patients who experienced OHCA in a public location. Of the 1639 public arrest cases, 131 (8%) were females and 1508 (92%) were males. Bystander CPR was provided to 47.3% of females and 42.6% of males (*p* = 0.29). The crude OR of provision of bystander CPR (female vs. male) was 1.21, 95% CI 0.85–1.73, *p* = 0.29. When adjusted for age, witness status, period of arrest, and initial rhythm, the analysis showed that female gender was associated with greater odds of provision of bystander CPR (adjusted OR 1.47, 95% CI 1.10–1.82, *p* = 0.04) (Table 3).

**Table 3:** Association between gender and bystander CPR-patients arrested in public location (*N* = 1639)

Variable	OR^a^	(95% CI^b^)	*P-*values
Female gender (crude)	1.21	0.85–1.73	0.291
Female gender (adjusted)	1.47	1.10–1.82	0.044
Age	1.01	0.01–1.02	0.022
Witnessed arrest	5.37	3.81– 7.57	< 0.001
Initial rhythm	1.78	1.43–2.23	< 0.001
Non-pandemic period	1.25	1.10–1.43	< 0.001

^a^*OR* Odds ratio

^b^*CI* Confidence intervals

## Discussion

We examined 4283 adults with non-traumatic OHCA from HMC OHCA registry in the State of Qatar and investigated sex differences in the provision of bystander CPR. Our crude analysis showed bystander CPR was less common in female gender; however, after adjustment for covariates, including arrest location, gender was no longer significant. Among the subgroup who arrested in public locations, our analysis revealed that females had greater odds of receiving bystander CPR than males. These results suggest that females are more likely to receive bystander CPR if their cardiac arrest occurs in public location. We believe this is an important finding and has rarely been reported previously. These findings indicate that the likelihood of receiving bystander CPR may be less related to gender itself but more related to differences in the locations at which females or males may have an OHCA. Further research in this area is required.

Notably, while our results suggest that females are more likely to receive bystander CPR if their cardiac arrest occurs in public location, we found that a larger proportion of females experiencing OHCA in private locations compared to males. In Qatar, there is a strong emphasis on family privacy and familial domains, perhaps leading to women spending longer time in the home environment. These societal dynamics could potentially explain a higher incidence of OHCA in private locations among females [24]. Measures such as community awareness and training for OHCA, along with the identification of high-risk groups and the provision of home monitoring devices, hold the potential to deliver early CPR and improve OHCA outcomes.

While our unadjusted results are in line with the findings of other studies [12, 13, 15, 17, 30, 31], our adjusted results are not consistent with other research that reported females had significantly lower odds of receiving bystander CPR in public locations [18] or reported no significant difference between the females and males in bystander CPR in public locations [10, 12, 30]. The reasons why females who arrested in public locations are more likely to receive bystander CPR than their male counterparts are not clear. It could be due to societal norms. In many cultures, including Middle Eastern culture, women are highly respected and valued members of society, which may motivate individuals to provide special care to them during a medical emergency. Further research is needed to determine whether cultural factors are at play.

Another important finding of our study is that out of all OHCA patients, only 34.2% received bystander CPR. This rate is relatively small in comparison with data from other countries that have shown higher proportions of OHCA cases receiving bystander CPR, bystander CPR rate is 43% in Japan [32], 48% in West Canada [12, 15], 66% in Seattle, USA [33], and 71% in Holland [17]. The reason for low bystander CPR rate in Qatar is not clear. It is possible that people are reluctant to act in an emergency and initiate CPR due to lack of training, low confidence, fear of legal liability, or fear of infection. Another possible contributing factor is that the male population in Qatar has a high proportion of migrant workers (81.2% of the total labor force) [34]. These workers are often employed in physically demanding and risky occupations, and they may be less likely to receive bystander CPR due to socioeconomic status [35]. More research is needed to establish a definitive link between these factors and the rate of bystander CPR in Qatar.

While the primary focus of this study is the provision of bystander CPR, it is important to acknowledge that the EMS system in Qatar, which includes treatment protocol and advanced interventions, could influence achievement of ROSC and overall survival rate [27, 36]. Nevertheless, our finding showed bystander CPR rate remains relatively low. To improve this, decision-makers should consider strategies such as increasing public awareness, providing more training opportunities, and addressing concerns related to legal liability. Implementing these steps could enhance the bystander CPR rate, potentially leading to better outcomes for OHCA victims. Previous studies have demonstrated that such campaigns effectively increase public resuscitation knowledge, raise awareness of the responsibility to help others, boost self-confidence to provide bystander CPR, and significantly improve bystander CPR and defibrillation rates, thereby enhancing the OHCA survival rate [37–41].

Interestingly, our results showed that the COVID-19 pandemic did not influence bystander resuscitation behavior. Despite the pandemic introducing various challenges like increasing personal health risk perceptions and the need for physical distancing, our study found no significant decline in CPR willingness. This contrasts with several other studies reporting reduced bystander CPR rates during the pandemic [42–44]. The difference in our findings may be attributed to the sociodemographic characteristics of Qatar’s population, mainly comprising younger individuals and men who tend to display fewer negative attitudes and behaviors [45]. It is possible that they performed CPR using safer measures, such as hands-only CPR, facemask protection, and following EMS dispatch phone instructions.

It is worth mentioning that our study found a lower incidence of cardiac arrest in females. This could be because the female population in Qatar is smaller (approximately one third vs. two thirds) compared to males, with the latter having a substantial representation of migrant workers with poorer socioeconomic status [22, 34]. This may contribute to the observed difference in OHCA rates. The lower incidence of OHCA in females might be also linked to the physiological factors including the protective effects of sex hormones, making them less susceptible to developing cardiac arrest [46].

While our study provides important insights into association between gender and bystander CPR in a Middle Eastern society, it is important to acknowledge its limitations. Firstly, our study analyzed data from Qatar, and therefore, the generalizability of our findings to other populations and regions may be limited. Secondly, as other observational studies, our study is vulnerable to unmeasured confounders. Data on some variables, such as comorbidities and event time, were incomplete in the dataset and therefore were not included in the analysis, which may have influenced our findings. Lastly, our dataset lacked information on whether there is a difference in response time between public and private arrests. However, our results did indicate a lower incidence of female arrests in public, which could be associated with longer response times and may have influenced the outcomes.

## Conclusions

Overall, OHCA baseline characteristics, including the provision of bystander CPR, were not in favor of females. Bystander CPR was less common in female gender; after adjustment for other covariates, including arrest location, we found no significant gender differences in provision of bystander CPR. Further subgroup analysis showed that among OHCA cases who experience OHCA in public location, female gender was associated with greater odds of bystander CPR. However, females were less likely to experience cardiac arrest in public locations.

## Data Availability

The data that support the findings of this study are available from the Hamad Medical Corporation (HMC) OHCA registry, but restrictions apply to the availability of these data, which were used under license for the current study, and so are not publicly available. Data are however available from the authors upon reasonable request and with permission of HMC.
